# Phylogeny, adaptive evolution, and taxonomy of *Acronema* (Apiaceae): evidence from plastid phylogenomics and morphological data

**DOI:** 10.3389/fpls.2024.1425158

**Published:** 2024-08-16

**Authors:** Lian Chen, Bo-Ni Song, Lei Yang, Yuan Wang, Yun-Yi Wang, Xueyimu Aou, Xing-Jin He, Song-Dong Zhou

**Affiliations:** Key Laboratory of Bio-Resources and Eco-Environment of Ministry of Education, College of Life Sciences, Sichuan University, Chengdu, China

**Keywords:** Apiaceae, *Acronema*, adaptive evolution, DNA barcoding, phylogeny, plastome, taxonomy

## Abstract

**Introduction:**

The genus *Acronema*, belonging to Apiaceae, includes approximately 25 species distributed in the high-altitude Sino-Himalayan region from E Nepal to SW China. This genus is a taxonomically complex genus with often indistinct species boundaries and problematic generic delimitation with *Sinocarum* and other close genera, largely due to the varied morphological characteristics.

**Methods:**

To explore the phylogenetic relationships and clarify the limits of the genus *Acronema* and its related genera, we reconstructed a reliable phylogenetic framework with high support and resolution based on two molecular datasets (plastome data and ITS sequences) and performed morphological analyses.

**Results:**

Both phylogenetic analyses robustly supported that *Acronema* was a non-monophyletic group that fell into two clades: *Acronema* Clade and East-Asia Clade. We also newly sequenced and assembled sixteen Acronema complete plastomes and performed comprehensively comparative analyses for this genus. The comparative results showed that the plastome structure, gene number, GC content, codon bias patterns were high similarity, but varied in borders of SC/IR and we identified six different types of SC/IR border. The SC/IR boundaries of *Acronema chienii* were significantly different from the other *Acronema* members which was consistent with the type VI pattern in the genus *Tongoloa*. We also identified twelve potential DNA barcode regions (*ccsA*, *matK*, *ndhF*, *ndhG*, *psaI*, *psbI*, *rpl32*, *rps15*, *ycf1*, *ycf3*, *psaI-ycf4* and *psbM-trnD*) for species identification in Acronema. The molecular evolution of Acronema was relatively conservative that only one gene (*petG*) was found to be under positive selection (*ω* = 1.02489).

**Discussion:**

The gene petG is one of the genes involved in the transmission of photosynthetic electron chains during photosynthesis, which plays a crucial role in the process of photosynthesis in plants. This is also a manifestation of the adaptive evolution of plants in high-altitude areas to the environment. In conclusion, our study provides novel insights into the plastome adaptive evolution, phylogeny, and taxonomy of genus *Acronema*.

## 1 Introduction

The genus *Acronema* Falconer ex Edgeworth., belonging to the family Apiaceae, is distributed in the high-altitude Sino-Himalayan region from E Nepal to SW China. This genus includes about 25 species in total, and 20 species are distributed in China (including 14 endemic). The members of the genus *Acronema* are morphologically distinguished from other genera of Apioideae mainly by the special long-linear or long-aristate apex of petals and thin stems with globose or tuberous roots (She et al., 2005a). However, several *Acronema* taxa hold acute or obtuse but not long-linear apex of petals, for example, *Acronema chinense* H. Wolff, *A. chinense* var. *humile* S. L. Liou & R. H. Shan, and *Acronema minus* (M. F. Watson) M. F. Watson & Z. H. Pan, and these features were also observed in plants of the genus *Sinocarum* H. Wolff ex R. H. Shan & F. T. Pu (She et al., 2005b), making them difficult to distinguish from the members of *Sinocarum*. Based on reviews of the type specimens and morphological evidence, Pimenov (2017) treated *Acronema chienii* R. H. Shan and *A. chienii* var. *dissectum* R. H. Shan as synonyms of *Tongoloa taeniophylla* H. Wolff, as well as treated *Acronema acronemifolium* (C. B. Clarke) H. Wolff as a synonym of *Pternopetalum molle* (Franch.) Hand.-Mazz. in his checklist of Chinese Umbelliferae. We noticed that traditional methods to distinguish these species were mainly based on their morphological features, whereas many above-mentioned species always exhibited varied morphological features of leaf division, bracteoles, and mericarps, leading to extremely difficult generic delimitation. Therefore, the generic limits of *Acronema* based on morphological characteristics face challenges, and re-evaluation of the generic limits of *Acronema* is urgently needed.

In addition, the *Acronema* species grow in the high-altitude Sino-Himalayan region, where their plants are thin and weak. Except for the flowering period, most species of this genus are difficult to identify in other periods, which severely hinders the morphological studies of these taxa and makes species identification difficult. In addition, morphological materials such as flowers, mericarps, and leaves are extremely lacking. Although previous studies have involved the karyotype, seeding structure, and morphology for the genus, which significantly improved our understanding of this taxonomically notorious group, sampling of this genus involved was very limited (especially for the Chinese endemics), which failed to address the phylogenetic relationships of the genus (Liu and Shan, 1980; Cauwet, 1982; Alexeeva et al., 2000; Pimenov et al., 2001; Pakenham, 2010; Kljuykov et al., 2014). Therefore, it is necessary to collect the species of the genus comprehensively and further clarify their interspecific boundaries.

Previously, a few molecular markers, including nuclear ribosomal DNA internal transcribed spacer (ITS), plastid DNA *rpl*16 and *rps*16 intron, have been used to study the phylogeny of *Acronema* (Zhou et al., 2008, 2009; Liu et al., 2019; Xiao et al., 2021; Zhou et al., 2023). For example, Zhou et al. (2008) performed a phylogenetic analysis based on 106 ITS sequences from 100 taxa of the Apiaceae subfamily Apioideae, which included two *Acronema* species (*Acronema astrantiifolium* H. Wolff and *Acronema schneideri* H. Wolff), proposed “*Acronema* Clade” for the first time, and suggested that the members of *Acronema* were located in *Acronema* Clade. Subsequently, Zhou et al. (2009) explored the phylogeny of Apiaceae based on ITS sequences and plastid DNA *rpl*16 and *rps*16 introns, which only involved four *Acronema* taxa [*A. astrantiifolium*, *Acronema paniculatum* (Franch.) H. Wolff, *A. schneideri*, and an unidentified *Acronema* species], and the phylogenetic relationships of the *Acronema* Clade also remained questionable. Based on the ITS sequences of four *Acronema* species [*A. astrantiifolium*, *Acronema muscicola* (Hand.-Mazz.) Hand.-Mazz., *A. paniculatum*, and *A. schneideri*], Liu et al. (2019) concluded that *Acronema* was a monophyletic group, which was closely related to the genus *Apium* L. and *Sinocarum*. Later, to investigate the phylogeny of the genus *Sinocarum*, Xiao et al. (2021) used ITS sequences and plastid DNA *rpl*16 and *rps*16 introns to perform phylogenetic analyses and found that the relationship between *Acronema* and *Sinocarum* was quite complicated. However, Zhou et al. (2023) suggested that *Acronema* was non-monophyletic based on ITS sequences, which contained nine *Acronema* taxa. Among them, *A. chienii* and *Acronema crassifolium* Huan C. Wang, X. M. Zhou & Y. H. Wang were located in the East-Asia Clade, while the other seven *Acronema* species (*A. astrantiifolium*, *A. muscicola*, *A. paniculatum*, *A. schneideri*, and three unidentified *Acronema* species) were located in the *Acronema* Clade and nested with the genus *Sinocarum*. Although these molecular studies have improved our understanding of this taxonomically notorious group, limited *Acronema* sampling, weak supports, and low resolutions of these phylogenetic trees could not provide valuable information for improvement in *Acronema* taxonomy. Therefore, additional molecular data are urgent to reconstruct phylogenetic relationships and re-evaluate the generic limits of *Acronema*.

Plastid is a semi-autonomous organelle, and the plastid genome (plastome) usually shows a typical quadripartite structure (two inverted repeat regions separated by a large single-copy region and a small single-copy region) (Ravi et al., 2008). Compared to the nuclear genome, the plastome is tiny, less prone to recombination, present in high copy numbers, and plays a considerable role in revealing variation among plant species in terms of both sequence and structure (Parks et al., 2009; Wicke et al., 2011); thus, it can be a useful tool for phylogenetic analyses and species identification (Rokas and Carroll, 2005; Philippe et al., 2011; Shaw et al., 2014; Schneider et al., 2021). With the rapid development of next-generation sequencing (NGS), it has become easier and cheaper to obtain the plastome data. Thus, plastomes have been widely used in phylogenetic analyses of angiosperms (Jansen et al., 2008; Daniell et al., 2016), especially for Apiaceae, a family with numerous taxonomic controversies. For example, Wen et al. (2021) used 74 protein coding sequences (CDSs) to reconstruct the phylogenetic framework for Apioideae and reveal the phylogenetic relationships among most major clades of Apioideae and discussed the possible evolutionary events by combining the plastome-based and nuclear-based phylogenetic trees. Then, Liu et al. (2022) described the plastome features of the genus *Peucedanum* L. (Apiaceae, subfamily Apioideae) and verified that plastomes were powerful tools for improving phylogenetic supports. Recently, Song et al. (2024a) revealed the patterns of plastome evolution and reestablished phylogenetic relationships and taxonomic framework of the genus *Sanicula* L. (Apiaceae, subfamily Saniculoideae) using 17 plastomes. Although there have been a large number of reports revealing the plastome features and phylogenetic relationship for multiple taxa of Apiaceae, the plastome data of the genus *Acronema* are extremely lacking, and only one complete plastome of *Acronema* (*A. schneideri*) has been reported previously (Xiao et al., 2021), which severely hinders the study of the genus. Thus, we collected 16 *Acronema* taxa and newly sequenced their plastomes.

In this study, our main aims were to 1) investigate the plastome features and infer the plastome evolution of *Acronema* plants, 2) select highly variable hotspot regions as potential DNA barcodes for species identification of *Acronema*, and 3) reveal the phylogenetic position of this controversial genus.

## 2 Material and methods

### 2.1 Materials, DNA extraction, and sequencing

**Figure 1:**
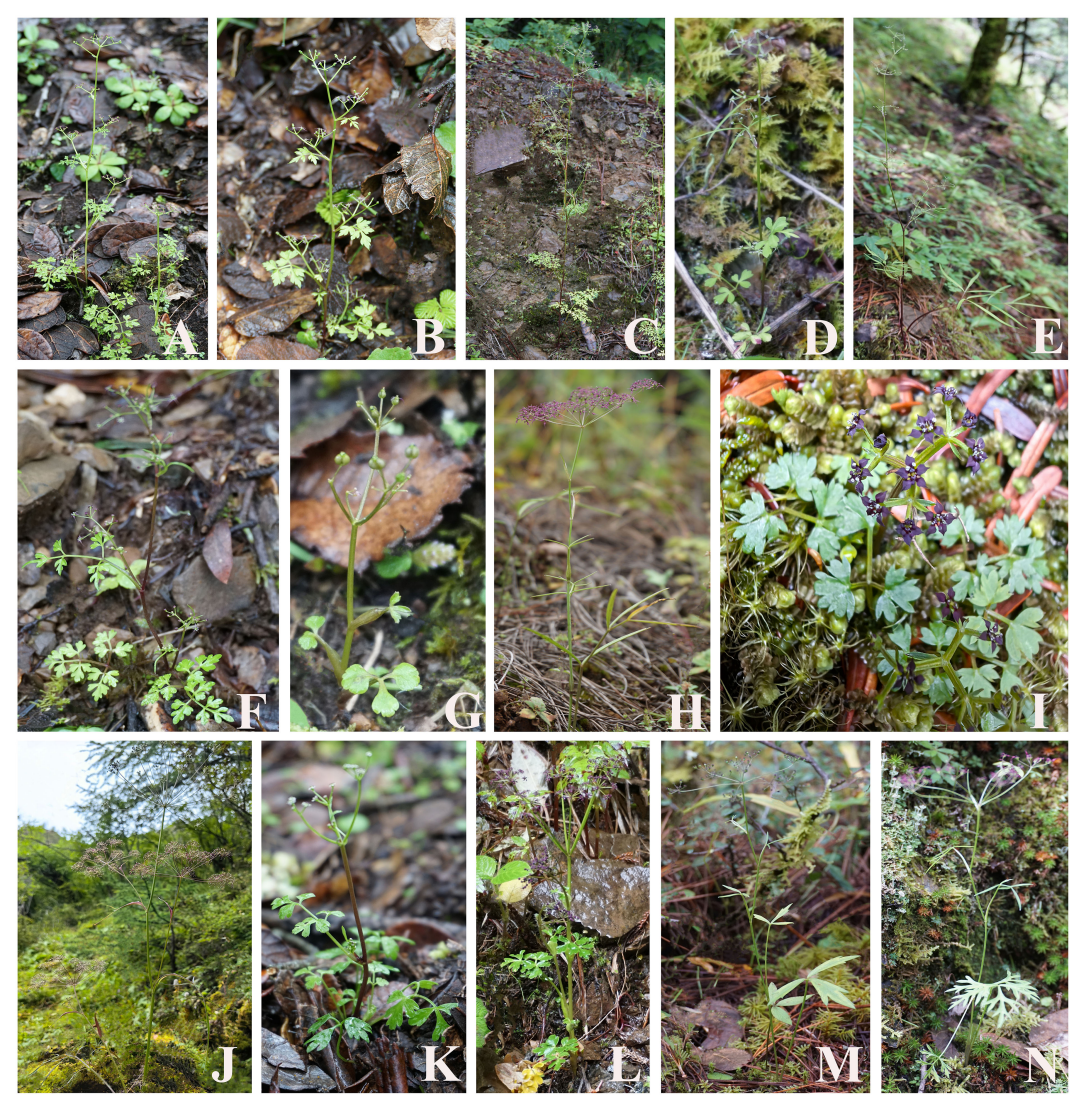
Illustrations of 14 species for Acronema in field: **(A)** *Acronema tenerum*, **(B)** *Acronema handelii*, **(C)** *Acronema paniculatum*, **(D)** *Acronema hookeri*, **(E)** *Acronema graminifolium*, **(F)** *Acronema commutatum*, **(G)** *Acronema muscicola*, **(H)** *Acronema schneideri*, **(I)** *Acronema minus*, **(J)** *Acronema chienii*, **(K)** *Acronema chinense* var. *humile*, **(L)** *Acronema crassifolium*, **(M)** *Acronema astrantiifolium*, and **(N)** *Acronema forrestii*.

All plant materials were collected from the field (**Figure 1**), and the fresh leaves were preserved in silica gel for further DNA extraction. The formal identification of these taxa was carried out by Professor Xing-jin He (Sichuan University). Vouchers were stored in the herbarium of Sichuan University (Chengdu, China) (**Supplementary Table 1**). Total DNA was extracted from silica-dried fresh leaves using the modified cetyltrimethylammonium bromide (CTAB) method (Doyle, 1987).

Complete plastomes of 16 *Acronema* taxa were sequenced on an Illumina NovaSeq platform at Personalbio (Shanghai, China), applying the paired-end 150-bp reads with an average insert size of 300–400 bp. Then, the obtained raw data were trimmed by removing adaptors, and low-quality reads were filtered using AdapterRemoval v2 (trimwindows = 5; minlength = 50) (Davis et al., 2013). Finally, high-quality reads with fastP v0.15.0 (-n 10 and -q 15) (Gu et al., 2018) were gained, yielding at least 10-GB clean reads for each species. A 30-μL amplification system was employed for ITS regions [2 μL extracted total DNA, 10 μL ddH_2_O, 15 μL Taq MasterMix (CWBio, Beijing, China), 1.5 μL of 10 pmol/μL forward primers, and 1.5 μL of 10 pmol/μL reverse primers]. The PCR cycling started at 94°C for 3 min to initialize denaturation, then at 94°C for 45 s to denaturation, 30 cycles of 45 s at 94°C, annealing at 55°C for 45 s and extension at 72°C for 45 s, and final extension at 72°C for 7 min, storage at 4°C (White, 1990). All PCR products were sequenced at Sangon Biotech (Shanghai, China). Finally, Geneious v9.0.2 (Kearse et al., 2012) was used to edit and gain the consensus sequence.

### 2.2 Plastome assembly, annotation, and comparison

NOVOPlasty v2.6.2 (K-mer = 39) (Dierckxsens et al., 2017) was used for *de novo* assembly of clean reads with default parameters, and *rbcL* sequence was extracted from *A. schneideri* (NC_064352) as seed. PGA software (Qu et al., 2019) was employed for the preliminary annotation of plastomes with *A. schneideri* (NC_064352) as the reference; then, start and stop codons were checked and manually corrected with Geneious v9.0.2 (Kearse et al., 2012). The online program OrganellarGenomeDRAW (OGDRAW) (Stephan et al., 2019) was used to draw the plastome map and alignment tool Mauve (Darling et al., 2010) to identify repetitive regions. All newly plastome data and ITS sequences were submitted to the National Center for Biotechnology Information (NCBI) under the accession numbers (**Supplementary Table 1**). The boundaries of SC/IRs were analyzed by IRscope (Amiryousefi et al., 2018) and then checked and manually adjusted in Geneious v9.0.2 (Kearse et al., 2012).

### 2.3 Repeat types and simple sequence repeat analysis

The four types of repeats [Forward (F), Reverse (R), Complement (C), and Palindromic (P)] were identified by the online program REPuter (Kurtz et al., 2001) with parameters set to hamming distance = 3 and minimal repeats size = 30; then, the number of each repeat was counted. Simple sequence repeats (SSRs) were identified by the web MISA (Beier et al., 2017), and parameters were set to 10, 5, 4, 3, 3, and 3 for mononucleotide, dinucleotide, trinucleotide, tetranucleotide, pentanucleotide, and hexanucleotide. Finally, the number of six types of SSRs was counted, and the frequency of their distribution in the SC/IR regions was calculated.

### 2.4 Codon usage bias, Ka/Ks analysis, and hotspot identification

The repetitive sequences were first removed, CDSs of less than 300 bp were eliminated, 53 shared CDS were obtained, and codon usage bias analysis was performed in MEGA6 (Tamura et al., 2013). Then, the heat map was drawn by TBtools (Chen et al., 2020). A total of 79 protein coding genes (PCGs) and 51 common intergenic regions were first extracted in Phylosuite v1.2.2 (Zhang et al., 2020) and aligned in MAFFT v7.221 (Katoh and Standley, 2013). DnaSP v5.1 (Librado and Rozas, 2009) was used to calculate nucleotide diversity values (Pi) and synonymous (Ks) and non-synonymous (Ka) nucleotide substitution rates.

### 2.5 Phylogenetic analysis

In order to identify the phylogeny of the genus *Acronema*, two datasets (PCG data and ITS sequences) were used to reconstruct the phylogenetic trees. A total of 85 plastome data and 84 ITS sequences from 30 genera of Apioideae were used to perform phylogenetic analyses, and *Bupleurum chinense* Franch. and *Bupleurum falcatum* L. were set as outgroups as referred to in the previous study (Wen et al., 2021). The 84 ITS sequences were straightway aligned with MAFFT v7.221 (Katoh and Standley, 2013) to gain the matrix. For plastome data, 79 commonly shared PCGs were extracted from complete plastomes with Phylosuite v1.2.2 (Zhang et al., 2020) and aligned by MAFFT v7.221 (Katoh and Standley, 2013), trimmed by trimAI (Capella-Gutiérrez et al., 2009), and then concatenated them as a matrix using Phylosuite v1.2.2 (Zhang et al., 2020). Phylogenetic analysis of the matrix was performed in two methods: maximum likelihood analysis (ML) and Bayesian inference (BI). ModelFinder (Kalyaanamoorthy et al., 2017) was employed to select the best model for analysis. For the ML analysis, RAxML v8.2.8 (Stamatakis, 2014) with 1,000 replicates was used to estimate the value of bootstrap support (BS) for each node, and the model GTRGAMMA was matched. Meanwhile, MrBayes v3.2.7 (Ronquist et al., 2012) was employed to BI analysis with the best-fit nucleotide substitution model (GTR + I + G) for a matrix of both datasets. Finally, the initial 25% of sampled data were discarded, and the remaining trees were obtained to yield the consensus tree and calculate posterior probabilities (PPs). The tree ML and BI phylogenetic analyses were edited in FigTree v1.4.2 (Rambaut and Drummond, 2015).

### 2.6 Morphological observation

We collected flowers and mature mericarp materials of 13 *Acronema* species from the field and kept them in formaldehyde–acetic acid–ethanol (FAA) fixing solution to soften and preserve before the anatomical observation. The petal of a flower was directly observed under a stereomicroscope (SMZ25, Nikon Cor., Tokyo, Japan) and then photographed for preservation. The mericarp morphological characteristics were also observed under a stereomicroscope (SMZ25, Nikon Cor., Tokyo, Japan), including mericarp shape, mericarp surface, dorsal side, commissure and transverse section, fruit rib shape and number, and vittae number. The description of mericarp terminology refers to the research of Kljuykov et al. (2004) and Ostroumova (2021).

## 3 Results

### 3.1 Features and comparison of *Acronema* plastomes

**Figure 2:**
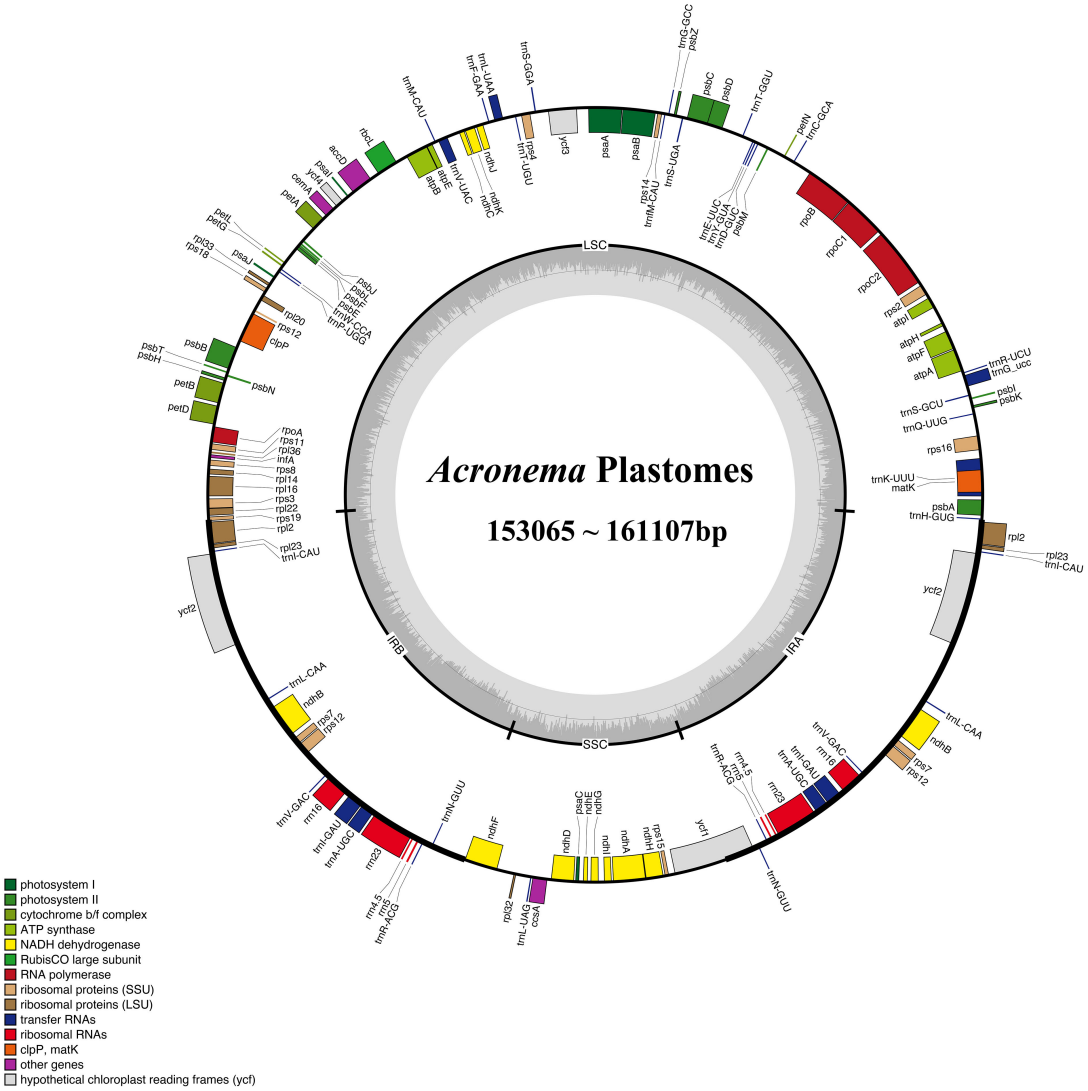
Gene map of the 16 *Acronema* plastome. The genes exhibited outside of the circle are transcribed clockwise, while those inside are counterclockwise. The genes belonging to different functional groups are color-coded.

We obtained complete plastomes from 16 *Acronema* species, and 15 plastomes were reported for the first time (except *A. schneideri* NC_064352). The total length of the plastomes ranged from 153,065 bp (*A. chinense* var. *humile*) to 161,107 bp [*Acronema tenerum* (Wall.) Edgew.]. All 16 plastomes had a typically quadripartite structure, including a large single-copy region (LSC; ranging from 81,687 to 85,801 bp), a small single-copy region (SSC; ranging from 16,568 to 17,855 bp), and a pair of reverse repeat regions (IRs; ranging from 25,479 to 30,725 bp). The total GC content ranged from 37.40% (*A. chinense* var. *humile*) to 37.72% [*Acronema hookeri* (C. B. Clarke) H. Wolff]. The gene composition was highly consistent among 16 plastomes, which possessed 113 unique genes, including 79 PCGs, four rRNA genes, and 30 tRNA genes (**Supplementary Table 1**; **Figure 2**).

**Figure 3:**
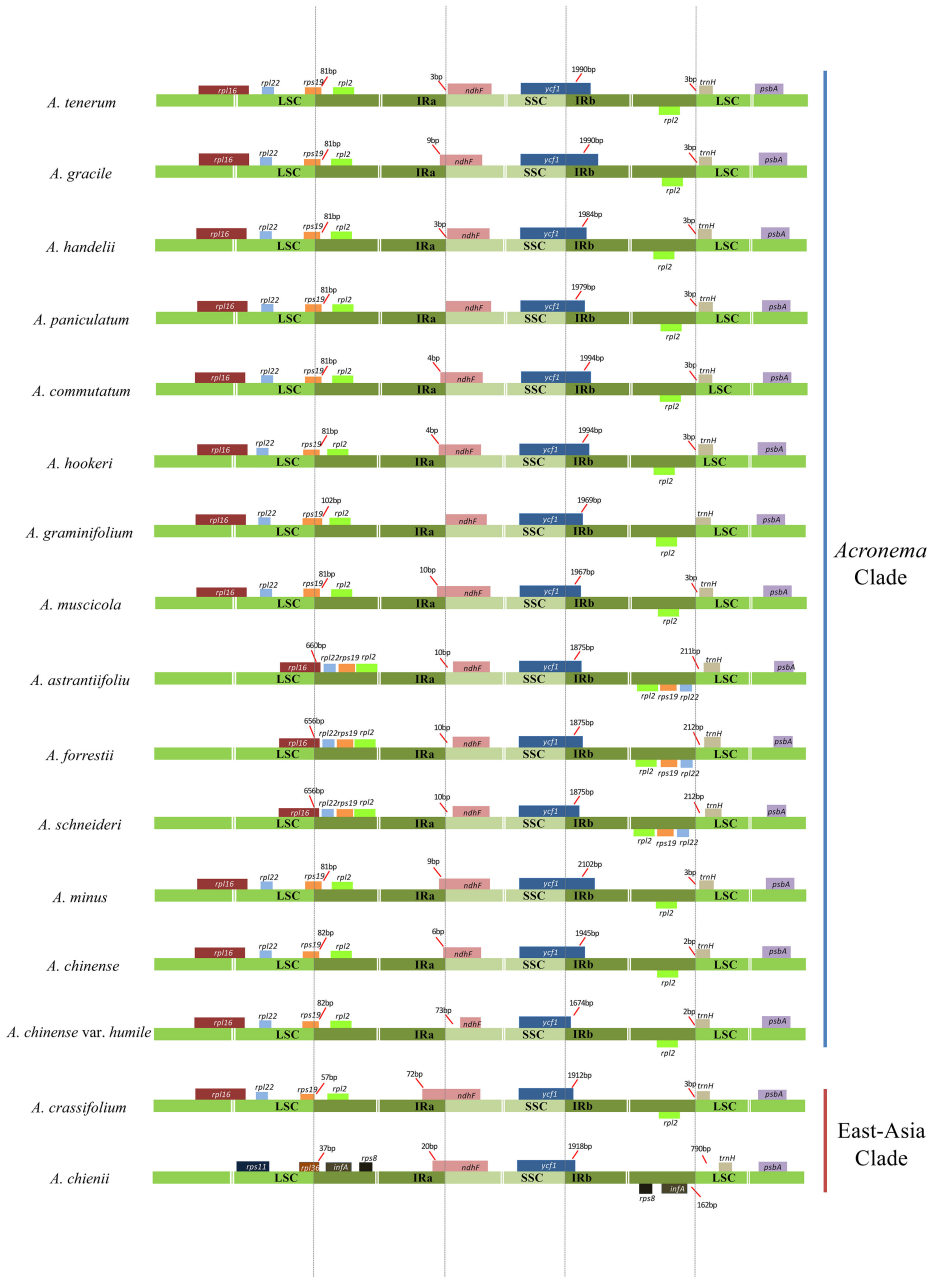
Comparison of the borders of the LSC, SSC, and IR regions among 16 *Acronema* plastomes. LSC, large long single copy; SSC, short single copy; IR, inverted repeat.

The boundary regions of LSC/IRs and SSC/IRs were also analyzed for these 16 plastomes (**Figure 3**). The boundary regions of LSC/IRa were divided into three types: type I represented that the gene *rpl*36 extended into IRa region with 37 bp in one plastome (*A. chienii*), type II represented that the gene *rpl*16 extended into IRa regions with 656–660 bp in three plastomes (*A. astrantiifolium*, *Acronema forrestii* H. Wolff, and *A. schneideri*), and type III represented that the gene *rps*19 extended into IRa regions with 57–102 bp in the remaining 12 *Acronema* plastomes. The IRa/SSC boundaries were also divided into three types: type IV showed no contraction or expansion of the gene *ndh*F in two plastomes [*A. paniculatum* and *Acronema graminifolium* (H. Wolff) S. L. Liou & R. H. Shan], type V showed that the gene *ndh*F was 3–73 bp away from the IRa/SSC borders in six plastomes (*A. astrantiifolium*, *A. chinense* var. *humile*, *A. forrestii*, *Acronema handelii* H. Wolff, *A. tenerum*, and *A. schneideri*), and type VI showed that the gene *ndh*F extended 4–72 bp into IRa regions in the remaining eight *Acronema* plastomes. The *ycf*1 genes, crossing the SSC/IRb borders, were located in the IRb regions with 1,674–2,102 bp. The IRb/LSC boundaries were located between the *rpl*2 gene and *trn*H gene in 12 plastomes and located between the *rpl*22 gene and *trn*H gene in three plastomes (*A. astrantiifolium*, *A. forrestii*, and *A. schneideri*), while in the *A. chienii* plastome, the IRb/LSC boundary was located between the genes *inf*A and *trn*H.

### 3.2 Repeat element analysis

**Figure 4:**
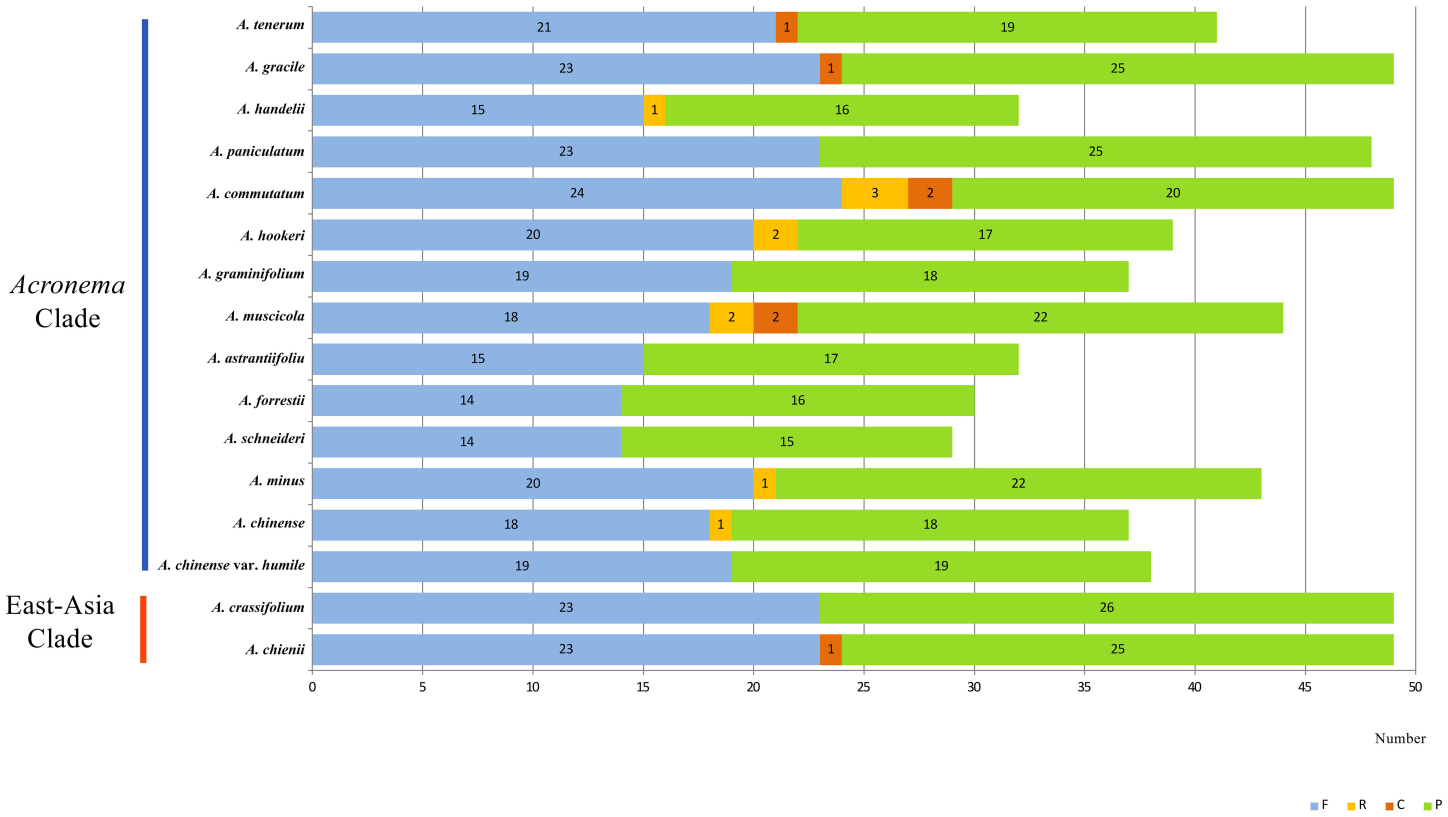
Dispersed repeats for 16 *Acronema* plastomes. Forward (F), Reverse (R), Complement (C), and Palindromic (P).

**Figure 5:**
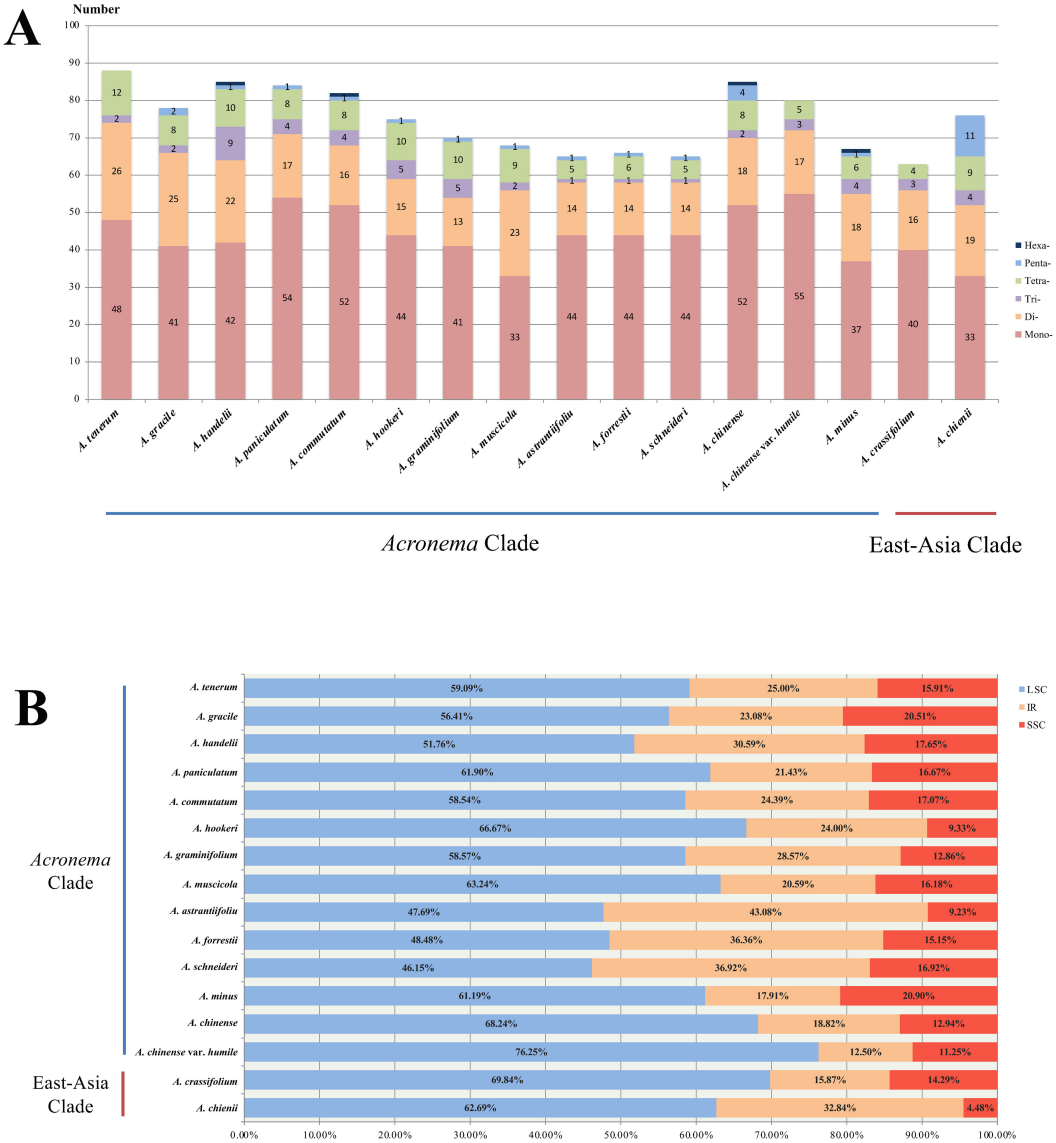
Analyses of simple sequence repeats (SSRs) in 16 *Acronema* plastomes. **(A)** Numbers of different repeat types. **(B)** Presence of SSRs in LSC, SSC, and IR. LSC, large long single copy; SSC, short single copy; IR, inverted repeat.

We investigated the dispersed repeats of 16 *Acronema* plastomes and found 29–49 repeat sequences (**Figure 4**; **Supplementary Table 2**). These dispersed repeats included Forward repeats (F; ranging from 14 to 24), Reverse repeats (R; ranging from 0 to 2), Complement repeats (C; ranging from 0 to 2), and Palindromic repeats (P; ranging from 15 to 26). In these 16 plastomes, *Acronema commutatum* H. Wolff and *A. muscicola* covered all four types of dispersed repeats: *A. chienii*, *A. chinense*, *Acronema gracile* S. L. Liou & R. H. Shan, *A. handelii*, *A. hookeri*, *A. minus*, and *A. tenerum* had three repeat types (F, R, P or F, C, P); seven plastomes (*A. astrantiifolium*, *A. chinense* var. *humile*, *A. crassifolium*, *A. forrestii*, *A. graminifolium*, *A. paniculatum*, and *A. schneideri*) possessed two repeat types (F and P). In addition, we also investigated SSRs, and the results showed that the total numbers of SSRs varied from 63 (*A. crassifolium*) to 88 (*A. tenerum*) (**Supplementary Table 3**). The Mono-, Di-, Tri-, and Tetra- repeats were found in 16 *Acronema* plastomes. Penta- repeat was absent in *A. chinense* var. *humile*, *A. tenerum*, and *A. crassifolium*. Hexa- repeat was present in four plastomes (*A. chinense*, *A. commutatum*, *A. handelii*, and *A. minus*). In these six repeat types, Mono- repeat accounted for the largest number of SSRs (ranging from 33 to 54) (**Figure 5A**). Most of these SSRs were distributed in the LSC regions (ranging from 46.15% to 76.25%), and SSC regions occupied the smallest proportion (ranging from 4.48% to 20.90%) (**Figure 5B**).

### 3.3 Molecular evolution and hotspot identification

**Figure 6:**
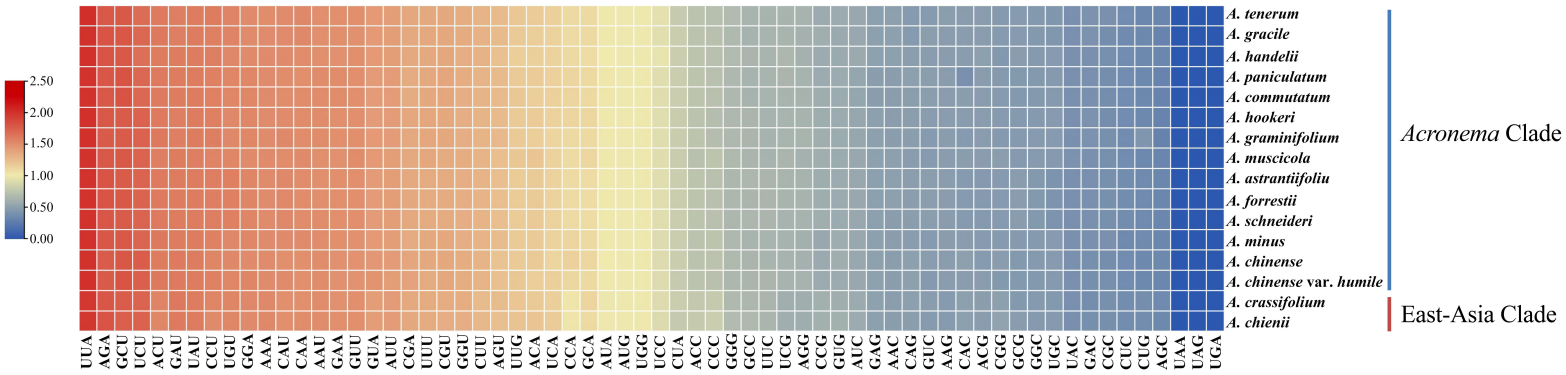
The RSCU values of all concatenated protein coding genes for 16 *Acronema* plastomes. Color key: the red values represent higher RSCU values, while the blue values indicate lower RSCU values. RSCU, relative synonymous codon usage.

To analyze the codon usage bias of *Acronema* species, CDSs less than 300 bp were first eliminated, and then 53 CDS were obtained and concatenated for each sample (**Figure 6**; **Supplementary Table 4**). These sequences varied from 71,568 to 74,614 bp and encoded 20,766–21,191 codons. Among these codons, Leu was encoded by the largest number of codons (2163–2240), while the least was Cys (217–223). Moreover, the codon usage bias was highly consistent among 16 plastomes. The value of relative synonymous codon usage (RSCU) was ≥1 for 32 codons, and most of them ended by A/U. Of these codons with RSCU ≥ 1, 1,604–1,672 codons encoded Leu, which was the most encoded amino acid, whereas the least was Cys (167–174).

**Figure 7:**
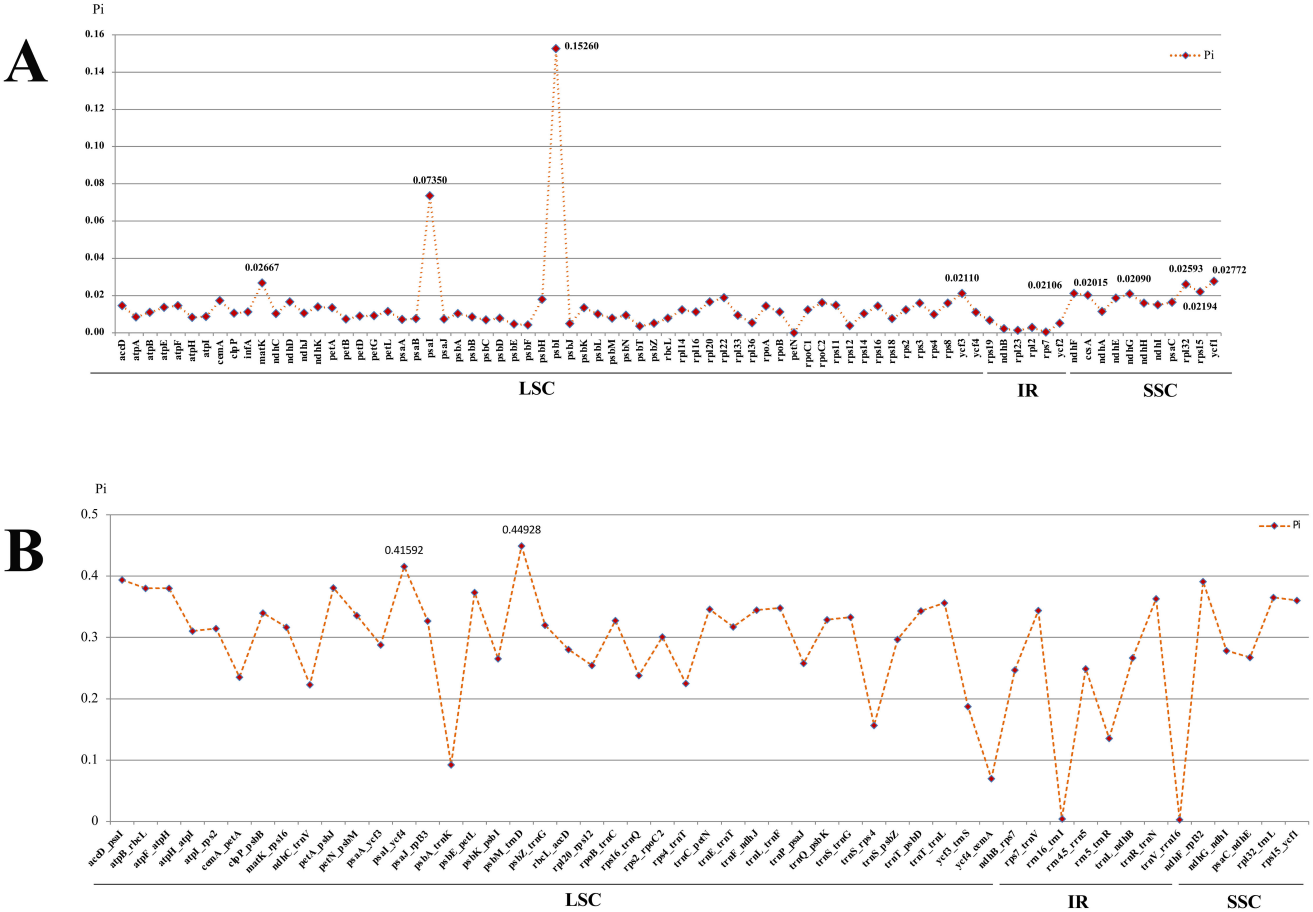
Comparative analysis of the nucleotide diversity (Pi) values among the 16 *Acronema* plastomes. **(A)** Protein coding genes. **(B)** Intergenic regions.

**Figure 8:**
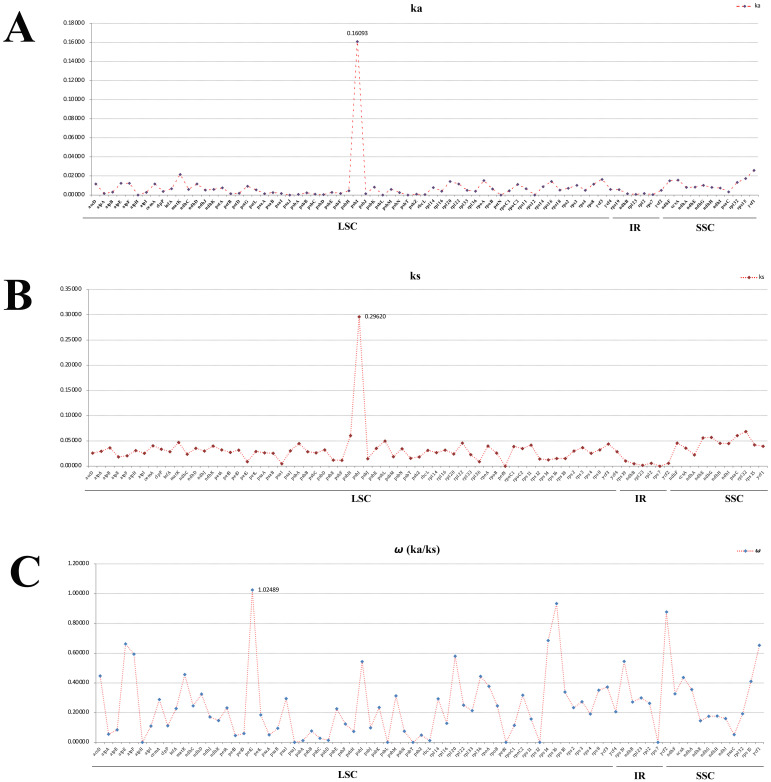
Average non-synonymous (Ka) **(A)**, synonymous (Ks) **(B)**, and Ka/Ks **(C)** values of protein coding genes. Genes *pet*G exhibited Ka/Ks values >1.

To identify the hotspots and select potential DNA barcoding regions for *Acronema*, 79 commonly PCGs and 51 common intergenic regions were used to calculate the nucleotide diversity (Pi) values, respectively (**Supplementary Table 5**; **Figure 7**). The Pi values among PCGs ranged from 0 (*pet*N) to 0.1526 (*psb*I) (**Figure 7A**), while Pi values within intergenic regions varied from 0.00293 (*trn*V*-rrn*16) to 0.44928 (*psb*M*-trn*D) (**Figure 7B**). Based on the sequence divergence, 12 mutation hotspot regions were selected as candidate DNA barcodes, containing 10 protein coding genes (*ccs*A, *mat*K, *ndh*F, *ndh*G, *psa*I, *psb*I, *rpl*32, *rps*15, *ycf*1, and *ycf*3; Pi > 0.02) and two intergenic regions (*psa*I*-ycf*4 and *psb*M*-trn*D; Pi > 0.4). Synonymous (Ks) and non-synonymous substitution (Ka) rates showed that the gene *psb*I obtained the highest value of both Ks and Ka (Ks = 0.29620; Ka = 0.16093). The values of ω (Ka/Ks) for 79 PCGs ranged from 0 (*atp*H, *psa*J, *psb*L, *psb*T, *pet*N, *rps*12, and *rps*7) to 1.02489 (*pet*G), and only one gene (*pet*G) had ω value greater than 1 (**Figure 8**; **Supplementary Table 6**). These findings indicated that only the *pet*G gene was under positive selection and that other genes were under purifying selection.

### 3.4 Phylogenetic analyses

**Figure 9:**
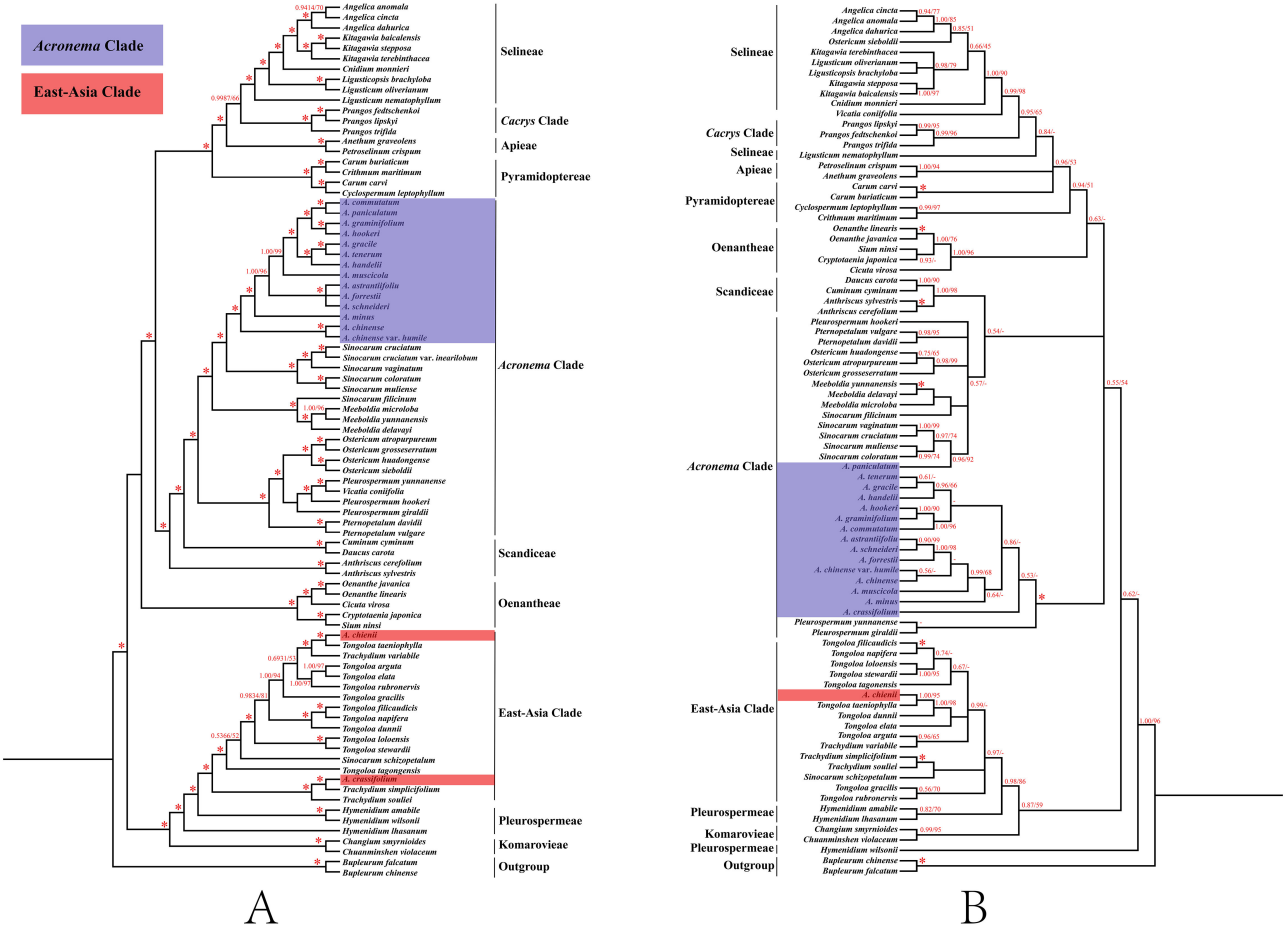
Phylogenetic trees constructed by maximum likelihood (ML) and Bayesian inference (BI). The bootstrap values (BS) of ML and posterior probabilities (PP) of BI are listed at each node. (*) represents the node with PP = 1.00/BS = 100, and (-) represents the node with PP < 0.50/BS < 50. **(A)** PCG tree. **(B)** ITS tree. PCG, protein coding gene; ITS, internal transcribed spacer.

In order to clarify the phylogenetic position of *Acronema*, we reconstructed the phylogenetic trees based on two datasets: 79 commonly shared PCGs of 85 complete plastomes and 84 ITS sequences (**Supplementary Tables 7**, **8**; **Figure 9**). Although little conflicts existed between the plastome phylogenetic trees (**Figure 9A**) and the ITS phylogenetic trees (**Figure 9B**), both strongly suggested that the non-monophyly of *Acronema* species, and these 16 *Acronema* species scattered in two clades: *Acronema* Clade and East-Asia Clade (**Figure 9**).

In the PCG-based tree, the analyses of ML and BI yielded that the topologies were highly identical, in which 16 *Acronema* taxa fell into two clades: *Acronema* Clade and East-Asia Clade. Among them, *A. chienii* and *A. crassifolium* belonged to East-Asia Clade. *A. chienii* and *T. taeniophylla* clustered together and then resolved as sister to *Trachydium variabile* H. Wolff (PP = 1, BS = 100). *A. crassifolium* clustered with *Trachydium simplicifolium* W. W. Sm. and formed a clade with *Trachydium souliei* H. Boissieu (PP = 1, BS = 100). The remaining 14 *Acronema* species clustered together and formed a clade with five *Sinocarum* species, belonging to *Acronema* Clade with high supports (PP = 1.00, BS = 100). *A. commutatum* gathered with *A. paniculatum*, as well as *A. graminifolium* gathered with *A. hookeri*, and then these four species formed a clade with high supports (PP = 1.00, BS = 100). *A. gracile* was more closely related to *A. tenerum*, and then, they clustered with *A. handelii* (PP = 1.00, BS = 100). Three species (*A. astrantiifolium*, *A. forrestii*, and *A. schneideri*) had a close affinity and clustered into a clade (PP = 1.00, BS = 100). *A. muscicola* and *A. minus* formed a single branch with robust supports (PP = 1.00, BS = 99; and PP = 1.00, BS = 100, respectively). *A. chinense* was resolved as a sister to its variety (*A. chinense* var. *humile*) (PP = 1.00, BS = 100) (**Figure 9A**).

In the ITS-based tree, the topologies resulting from ML and BI analysis were also identical. The phylogenetic positions of most species were consistent with the PCG-based phylogenetic trees, but there was little conflict. For example, 1) *A. crassifolium* clustered with *T. simplicifolium* (PP = 1.00, BS = 100), belonging to the East-Asia Clade in the PCG-based phylogenetic tree, while the former formed a single clade in the ITS-based tree with weak supports, belonging to the *Acronema* Clade (PP = 0.53, BS = 43). 2) *A. paniculatum* was sister to *A. commutatum* in the PCG-based phylogenetic tree (PP = 1.00, BS = 100), whereas the former made a clade with four *Sinocarum* species in the ITS-based phylogenetic tree (PP = 0.96, BS = 92) (**Figure 9B**).

### 3.5 Morphological characteristics

**Figure 10:**
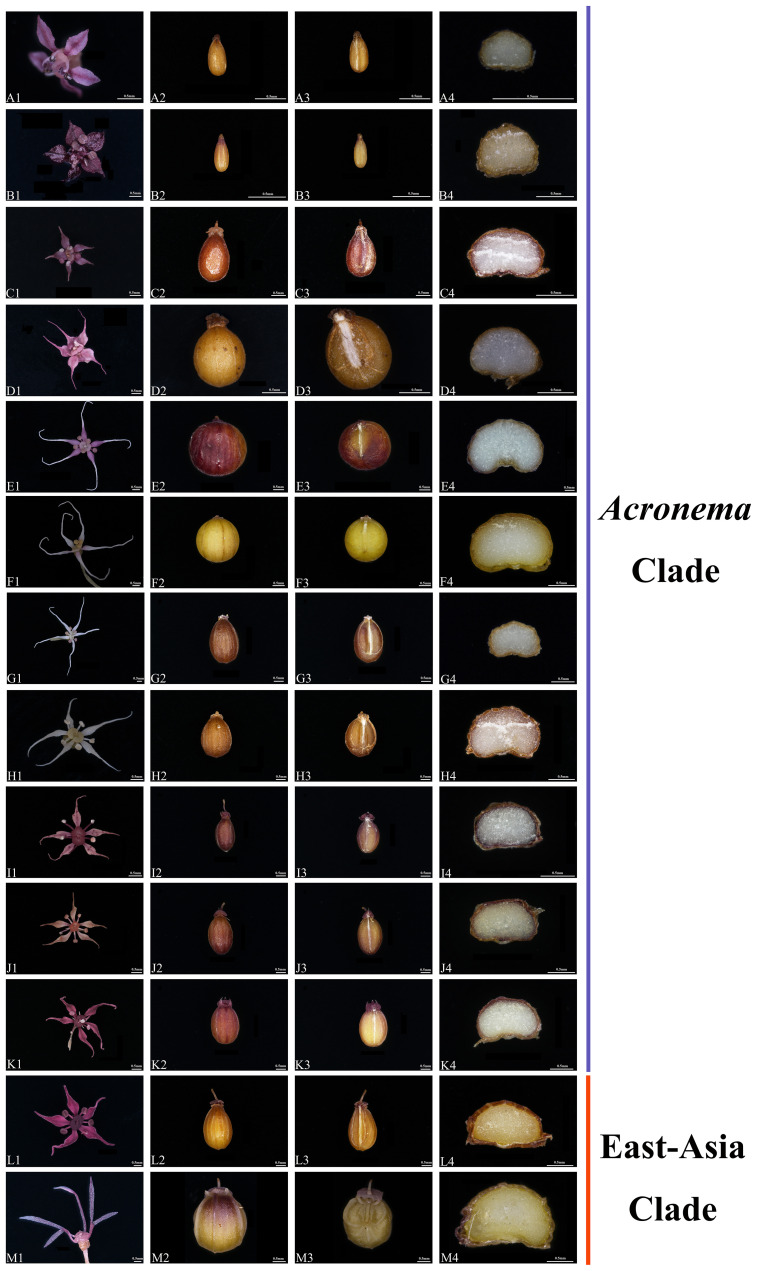
The flower and mericarp morphology of 13 *Acronema*. **(A)** *Acronema chinense* var. *humile*. **(B)** *Acronema minus*. **(C)** *Acronema tenerum*. **(D)** *Acronema muscicola*. **(E)** *Acronema paniculatum*. **(F)** *Acronema graminifolium*. **(G)** *Acronema commutatum*. **(H)** *Acronema hookeri*. **(I)** *Acronema schneideri*. **(J)** *Acronema forrestii*. **(K)** *Acronema astrantiifolium*. **(L)** *Acronema crassifolium*. **(M)** *Acronema chienii*. 1, petal; 2, dorsal side; 3, commissure; 4, transverse section. Scale = 0.5 mm.

We observed the morphology of petals and mature mericarps (dorsal side, commissure, and transverse section) of 13 *Acronema* species (**Table 1**, **Figure 10**). All mericarps are glabrous and slightly compressed dorsally and obtain five filiform ribs, which are small in most of the samples; commissure narrow, with endosperm almost flat or slightly groove on commissural side; transverse section sub-pentagon or sub-semicircular, vittae present or obsolete; petals ovate to ovate-lanceolate, apex acute, obtuse, liner, or long-liner. In detail, *A. chinense* var. *humile* and *A. minus* are similar in flower and mericarp morphology: oblong-ellipsoid mericarp and a transverse section showing sub-pentagon; vallecular vittae solitary and commissural vittae 2; ovate petals with short-acute apex, but not long-linear; meanwhile, the remains (except *A. chienii*) all show liner or long-liner apex of petals. *A. tenerum* (the type species of *Acronema*) shows a liner apex of petals and mericarp broad-ovoid, and the transverse section also shows a sub-pentagon; vallecular vittae solitary and commissural vittae obsolete. *A. graminifolium*, *A. muscicola* and *A. paniculatum* all have long-liner apex of petals and ovoid-orbicular mericarps, but vitta of *A. muscicola* obsolete, and the petals rhombic-ovate, while the other two species obtain solitary vallecular vittae and two commissural vittae, and ovate-lanceolate or narrow-lanceolate petals. *A. commutatum* and *A. hookeri* have narrow-lanceolate petals and apex long-liner; broad-ovoid mericarps with two to three vallecular vittae and 4 commissural vittae, but the latter is densely papillate on the apex of petals. *A. astrantiifolium*, *A. forrestii*, and *A. schneideri* are mainly distinguished by leaf morphology, but they are very similar in flower and mericarp morphology: the petals are ovate-lanceolate with liner and papillate apex and usually show purple-red; mature mericarps long-ovoid with vallecular vittae solitary or obsolete and commissural vittae 2. A*. crassifolium* is distinguished mainly by leathery leaves, and the petals are purple-red with liner apex and densely papillate, vittae well-developed, vallecular solitary, and commissural vittae 2. A*. chienii* has long-obovate petals and obtuse-acute apex, but not liner, and the mericarp is broadly ovate with a cordate base, vallecular vittae 2–3, and commissural 4, which is distinguished clearly from the other members of *Acronema*.

**Table 1:** The flower and mericarp morphology of 13 *Acronema*.

Taxon	Flower			Mericarp			Figure
	Petal	Apex	Shape	Transverse section	Rib	Vittae	
*Acronema chinense* var. *humile*	Ovate	Short-acute	Oblong-ellipsoid	Sub-pentagon, commissure flat	5, filiform	Vallecular solitary, commissural 2	**Figure 10A**
*Acronema minus*	Ovate	Short-acute	Oblong-ellipsoid	Sub-pentagon, commissure flat	5, filiform	Vallecular solitary, commissural 2	**Figure 10B**
*Acronema tenerum*	Ovate	Liner	Broad-ovoid	Sub-pentagon, commissure flat	5, filiform	Vallecular solitary, commissural obsolete	**Figure 10C**
*Acronema muscicola*	Rhombic-ovate	Long-liner	Ovoid-orbicular	Sub-pentagon, commissure flat	5, filiform	Obsolete	**Figure 10D**
*Acronema paniculatum*	Narrow-lanceolate	Long-liner	Ovoid-orbicular	Sub-pentagon, slightly groove on commissure	5, filiform	Vallecular solitary, commissural 2	**Figure 10E**
*Acronema graminifolium*	Ovate-lanceolate	Long-liner	Ovoid-orbicular	Sub-pentagon, commissure flat	5, filiform	Vallecular solitary, commissural 2	**Figure 10F**
*Acronema commutatum*	Narrow-lanceolate	Long-liner	Broad-ovoid	Sub-pentagon, slightly groove on commissure	5, filiform	Vallecular vittae 2–3, commissural vittae4	**Figure 10G**
*Acronema hookeri*	Narrow-lanceolate	Long-liner, papillate	Broad-ovoid	Sub-pentagon, slightly groove on commissure	5, filiform	Vallecular vittae 2–3, commissural vittae4	**Figure 10H**
*Acronema schneideri*	Ovate-lanceolate	Liner, papillate	Long-ovoid	Sub-pentagon, commissure flat	5, filiform	Vallecular solitary/obsolete, commissural 2	**Figure 10I**
*Acronema forrestii*	Ovate-lanceolate	Liner, papillate	Long-ovoid	Sub-pentagon, commissure flat	5, filiform	Vallecular solitary/obsolete, commissural 2	**Figure 10J**
*Acronema astrantiifolium*	Ovate-lanceolate	Liner, papillate	Long-ovoid	Sub-pentagon, commissure flat	5, filiform	Vallecular solitary/obsolete, commissural 2	**Figure 10K**
*Acronema crassifolium*	Ovate-lanceolate	Liner, papillate	Broad-ovoid	Sub-pentagon, commissure flat	5, filiform	Vallecular solitary, commissural 2	**Figure 10L**
*Acronema chienii*	Long-obovate	Obtuse-acute	Broad-ovoid, base cordate	Sub-semicircular, commissure flat	5, filiform	Vallecular vittae 2–3, commissural 4	**Figure 10M**

## 4 Discussion

### 4.1 Comparison of plastomes in *Acronema*

All *Acronema* plastomes displayed a typical quadripartite structure (one LSC region, one SSC region, and a pair of IR regions), which was also found in other plastomes of Apiaceae plants (Lei et al., 2022; Gui et al., 2023; Peng et al., 2023; Song et al., 2024a). In addition, *Acronema* plastomes were highly conserved in size, GC content, gene content and order, the patterns of codon bias, and SSRs. The situation of gene loss (pseudogenization or deletion) usually occurs during plastome evolution (Nguyen et al., 2015; Sun et al., 2020), and in this study, the gene *ycf*15 was missing in all plastomes, which also existed in other genera of Apiaceae (Shin et al., 2016; Liu et al., 2022; Gui et al., 2023). The phenomenon may occur independently during the evolution of plants. Thus, it may not provide us with valid phylogenetic signal. However, we detected some diversity in the 16 *Acronema* plastomes, with the most obvious being the SC/IR border. We identified six different types of SC/IR border, of which type I represented that the gene *rpl*36 extended into the IRa region with 37 bp only in *A. chienii*, which was consistent with the type VI pattern found by Gui et al. (2023) in the genus *Tongoloa*. Therefore, our plastid phylogenomic analyses further implied the non-monophyly of the *Acronema* genus.

### 4.2 Molecular evolution and DNA barcode identification

Codon is a useful and powerful signal in the plastome evolution and can be influenced by various factors (Mitreva et al., 2006; Parvathy et al., 2022). In this study, the codon usage bias patterns were similar among 16 *Acronema* samples, which revealed the conservation of *Acronema* plants in molecular evolution. In the analysis of synonymous and non-synonymous substitution, the resulting ω (Ka/Ks) is commonly used to indicate purifying selection (ω < 1), neutral evolution (ω = 1), and positive selection (ω > 1) of plastome genes that occur during the evolution (Yang and Nielsen, 2002; Ivanova et al., 2017). In this study, the value of ω (Ka/Ks) ranged from 0 to 1.02489, and 78 PCGs were under purifying selection (ranging from 0 to 0.93272), while only the *pet*G gene was found to be under positive selection (ω = 1.02489). The gene *pet*G is closely related to photosynthesis, which plays an important role in the synthesis of the key protein that promotes electron transfer between Photosystems I and II. Also, organic compounds such as sugars synthesized by photosynthesis can increase the concentration of cell fluid, which is beneficial for plants to resist low temperatures and obtain water in high-altitude environments (Schuster et al., 2020). Due to the fact that *Acronema* plants grow in damp and dense forests of high altitude, they often lack adequate natural light, clod, and aridification. Thus, in order to adapt to the ecological environment better, it is necessary to improve photosynthetic efficiency during evolution. This is a manifestation of the adaptive evolution of plants in high-altitude areas to the environment.

Species identification of the genus *Acronema* has always been a major challenge due to slender plants and the diverse morphological variation of vegetative organs such as leaves. With the rapid development of DNA barcoding technology, DNA barcode is now an effective tool to assist in the classification of species (Li et al., 2015; Coissac et al., 2016), especially for those controversial taxa (Hajibabaei et al., 2006; Kim et al., 2012; Liu et al., 2018; Xie et al., 2019). Also, in the Apiaceae plants, DNA barcodes were successfully identified in many taxonomic confusing species (Jiang et al., 2022; Liu et al., 2022; Song et al., 2023; Song et al., 2024b). In this study, we identified 12 mutation hotspot regions from 16 *Acronema* plastomes, including 10 PCGs (*ccs*A, *mat*K, *ndh*F, *ndh*G, *psa*I, *psb*I, *rpl*32, *rps*15, *ycf*1, and *ycf*3) and two intergenic regions (*psa*I*-ycf*4 and *psb*M*-trn*D). In these regions, *mat*K is a universal DNA barcode (CBOL Plant Working Group, 2009; Hollingsworth, 2011a, 2011b; Hollingsworth et al., 2016); *ccs*A, *ndh*F, *psa*I, *ycf*1, and *rps*15 are also identified in other genera of Apiaceae (Ren et al., 2020; Jiang et al., 2022; Liu et al., 2022; Song et al., 2023). The four high variable regions (*psa*I, *psb*I, *psa*I-*ycf*, and *psb*M-*trn*D) were specific to the genus *Acronema*, and therefore, they may be able to serve as specific potential DNA barcoding regions to distinguish *Acronema* species. These findings provide a valuable reference for further developing DNA barcodes for *Acronema*.

### 4.3 Phylogenetic relationship

The conflicts between plastome-based and ITS-based phylogenies often occurred in other genera of Apiaceae (Ren et al., 2020; Wen et al., 2021; Lei et al., 2022; Gui et al., 2023; Song et al., 2024b), and our study was no exception. *A. paniculatum* was sister to *A. commutatum* in the PCG-based phylogenetic tree, whereas the former made a clade with four *Sinocarum* species in the ITS phylogenetic tree with high supports (PP = 1.00, BS = 100; PP = 0.96, BS = 92). The habitats of *Acronema* and *Sinocarum* species are similar, and morphologically, *A. paniculatum* is similar to *Sinocarum* species in broad-ovate leaves that 2–3-pinnate with pinnae petiolules, and the leaves are upper linear but distinctly different by the tuberous root and ovate-lanceolate petals with long-linear apex of *A. paniculatum*, which is typical of *Acronema* members. Meanwhile, *A. crassifolium* (Wang et al., 2013) clustered with *T. simplicifolium* (PP = 1.00, BS = 100), belonging to the East-Asia Clade, while the former formed a single clade in the ITS-based tree with weak supports, belonging to the *Acronema* Clade (PP = 0.53, BS = 43). It indicated that there was a nuclear–plastome conflict in the phylogenetic position of *A. crassifolium*. The ITS-based phylogenetic relationship in the study of Zhou et al. (2023) showed that *A. crassifolium* belonged to the East-Asia Clade and was closely related to the genus *Trachydium* Lindl. with strong support, which was accordant with the result derived from plastome data in the present study but was not consistent with the ITS-based tree. Furthermore, *A. crassifolium* and *T. simplicifolium* grow in alpine meadows or gravel slopes at an altitude of 2,700–4,000 m. Both species have similar features, such as conical roots and ternate leaves with dark purple abaxial surfaces. However, the unique features that distinguish *A. crassifolium* from *T. simplicifolium* are the glabrous stem and the smooth surface of the mericarp, while it is scattered-tuberculate for *T. simplicifolium*, and the apex of petal of *A. crassifolium* is the liner, which coincides with *Acronema* species. Thus, we need to collect more populations and individuals for further study to clarify the phylogenetic position and taxonomic status of *A. crassifolium*. Members of *Acronema* grow in the dense forests, alpine meadows, or gravel slopes of the high-altitude Sino-Himalayan region, which have geographic sympatric distribution and reproductive compatibility with some other genera of Apiaceae, such as *Sinocarum*, *Trachydium*, and *Tongoloa*. Furthermore, the tectonic uplift of the Qinghai-Tibet Plateau (QTP) contributes to the diversity of species in this area, and arid alpine habitats with strong geographic isolation may lead to hybridization, introgression, incomplete lineage sorting (ILS), or chloroplast capture events during evolution (Folk et al., 2017; Mao et al., 2021), which may cause the generation and nesting of similar traits among related genera (Ashworth et al., 2015; Chen et al., 2023) and further result in the nuclear–plastome inconsistency. Therefore, further study is needed to identify the cause of the nuclear–plastome conflict in *Acronema*.

We clarified the taxonomic position of species *A. chienii* based on the phylogenetic analyses and morphological characteristics. Both the PCG-based and ITS-based phylogenetic trees robustly supported that *A. chienii* scattered into the East-Asia Clade and was distant from *A. tenerum* (type species of *Acronema*), but it was sister to *T. taeniophylla* with high supports (PP = 1.00, BS = 100; PP = 1.00, BS = 95). In a previous study, Zhou et al. (2023) also found that *A. chienii* is located in the East-Asia Clade. Pimenov (2017) treated *A. chienii* as a synonym of *T. taeniophylla* based on reviews of the type specimens and morphological evidence. Our phylogenetic analyses also supported that *A. chienii* should be transferred into *Tongoloa*. From the morphological evidence, *A. chienii* has some shared morphological features with the *Tongoloa* members, for example, stout and conic root, obtuse-acute apex of petals, and cordate base of mericarp, which is clearly distinguished from other *Acronema* species. Therefore, we supported that treating *A. chienii* as a synonym of *T. taeniophylla* was reasonable based on our molecular data and morphological evidence.

So far, except for the research by Zhou et al. (2023), other molecular phylogenetic studies based on a few molecular markers strongly supported the monophyly of the *Acronema* species (Zhou et al., 2008; Liu and Shan, 1980; Xiao et al., 2021; Zhou et al., 2009). In the current study, we conducted phylogenetic analyses for *Acronema* using plastome data and ITS sequences. Unfortunately, both datasets failed to recognize *Acronema* as a monophyletic group, and the 16 *Acronema* species fell into *Acronema* Clade and East-Asia Clade. Compared to previous phylogenetic studies employing a small number of DNA fragments or ITS sequences, our phylogenetic analyses based on 79 commonly shared PCGs of complete plastomes yielded a robust phylogenetic framework for 16 *Acronema* members. Thus, our phylogenetic framework was considered more reliable and convincing. Also, our morphological characteristics support the results of phylogenetic trees, too. For example, 14 *Acronema* species were located in the *Acronema* Clade in the PCG-based trees, and these species obtain similar morphological characteristics: thin stem, tuberous or globose root, acute, liner, or long-liner apex of petal and glabrous mericarps with five filiform ribs. Meanwhile, *A. chienii* and *A. crassifolium* were located in the East-Asia Clade in the PCG-based trees, but the morphological characteristics are clearly distinguished from those of *Acronema* species located in the *Acronema* Clade, especially their stout and conic root. Thus, our phylogenetic analyses and morphological evidence strongly support that *Acronema* is a non-monophyletic group.

## 5 Conclusion

The present study is the first attempt to use plastome data to comprehensively investigate the plastome characteristics and infer the phylogeny of *Acronema*. In this study, we newly sequenced and assembled 16 *Acronema* plastomes. Our results revealed that *Acronema* plastomes were highly conserved in structure, size, GC content, gene content and order, the patterns of codon bias, and SSRs. Nevertheless, 12 highly variable regions were still selected as potentially strong DNA barcodes for species identification in *Acronema*. Additionally, the molecular evolution of *Acronema* was relatively conservative, and only one gene (*pet*G) was found to be under positive selection, which may be related to adapting to the high-altitude environment. Furthermore, both phylogenetic trees based on PCGs data and ITS sequences strongly suggested the non-monophyly of *Acronema* species, which was further justified by our plastid phylogenomic analyses and morphological features. In summary, our study provided a reliable framework for *Acronema* and improved the taxonomic system of the Apiaceae family.

## Data Availability

The datasets presented in this study can be found in online repositories. The names of the repository/repositories and accession number(s) can be found in the article/**Supplementary material**.
